# High HIV prevalence and associated risk factors among transgender women in China: a cross‐sectional survey

**DOI:** 10.1002/jia2.25417

**Published:** 2019-11-15

**Authors:** Hongjing Yan, Wenjing Xiao, Yunting Chen, Yuanfang Chen, Jessica Lin, Zihan Yan, Erin Wilson, Willi McFarland

**Affiliations:** ^1^ Section of AIDS Control and Prevention Jiangsu Provincial Center for Disease Control and Prevention Nanjing China; ^2^ Nanjing Medical University Nanjing China; ^3^ Center for Public Health Research San Francisco Department of Public Health San Francisco CA USA; ^4^ University of California Berkeley Berkeley CA USA; ^5^ Department of Epidemiology and Biostatistics University of California San Francisco San Francisco CA USA

**Keywords:** transgender women, HIV prevalence, risk factors, China, sex work, sexual behaviour

## Abstract

**Introduction:**

Transgender women may face the highest prevalence of HIV of any population, experiencing a disproportionate burden of disease frequently confirmed in surveys throughout the developing and developed world. However, few studies have been conducted specifically for transgender women in China. This study aimed to measure HIV prevalence and explore risk factors for infection in a diverse sample of Chinese transgender women to help advocate for prevention and care interventions for this population.

**Methods:**

From July 2018 to May 2019, we adapted a respondent‐driven sampling (RDS) approach to recruit a diverse sample of 250 transgender women through chains of peer referrals in two cities of eastern China, Nanjing and Suzhou. Eligible participants (i.e. 18 years of age or older, living in Jiangsu province and assigned male sex at birth but currently self‐identified as a gender different from male) completed a self‐administered questionnaire on a mobile phone to collect demographic characteristics and risk behaviours and underwent HIV testing.

**Results and discussion:**

The survey sample was young (82% under age 35 years), with 28.8% having a university degree, 39.2% reporting work at entertainment venues, 47.6% ever having taken hormones and 6.4% being diagnosed with an STI in the last year. One in five (20.8%) reported having engaged in sex work. HIV prevalence was 14.8% (95% CI 10.6 to 19.8), with 75.6% of those testing HIV positive reporting they were already aware of their serostatus. In multivariate analysis, HIV prevalence was significantly higher among transgender women above the age of 24 years, those who work at entertainment venues, who never have taken hormones, and who had been diagnosed with an STI in the last year.

**Conclusions:**

The prevalence of HIV among transgender women in our study, at 14.8%, is among the highest detected in any population in eastern China. Chinese transgender women may therefore follow the disparity in the burden of HIV noted worldwide. Data support policies to prioritize transgender women for HIV testing outreach, for in‐depth research to better understand the specific drivers of infection in this population, and for trans‐friendly HIV care and prevention programmes to address their specific needs.

## 1 Introduction

Transgender women, people who were assigned a “male” sex at birth but identify as a gender other than man, bear the highest burden of HIV of any population worldwide. A global meta‐analysis places HIV prevalence among transgender women at 19.1%, with the odds of infection 48.8 times that of other adults 1. Even in sub‐Saharan Africa, the region of the world with the highest burden of HIV infection among the general population, transgender women have significantly higher HIV prevalence 2. In the few Asian countries with data on transgender women, surveys have also demonstrated high prevalence of HIV, for example, in Indonesia (26.1%), Thailand (12.5%), Vietnam (18.0%) and Cambodia (5.7%) 1, 3, 4.

International research indicates that stigma and discrimination towards transgender women leads to biological, behavioural, social, cultural and economical vulnerabilities for HIV that vary across countries and regions 5, 6. For example, in several African countries, depression, condomless receptive anal sex, law enforcement stigma and violence were significantly associated with HIV 2. In the United States and Peru 7, race, multiple sexual partners, condomless receptive anal or vaginal sex, non‐prescribed injection of hormones or drugs, sharing drug paraphernalia, engaging in commercial sex work and history of sexually transmitted infections (STI) were associated with HIV 7, 8, 9. In Southeast Asia, age, alcohol use, amphetamine use, sex with male sex workers and dressing as a woman all the time were risk factors for HIV 3, 4. However, data on HIV prevalence and correlates of HIV infection among transgender women in China are scant.

Since the first AIDS case was reported in 1985 in China, the prevailing pattern of HIV transmission has evolved through different vulnerable populations, often reaching substantial prevalence long before being detected 10. For example, in some regions of China, HIV initially spread rapidly among people who inject drugs through sharing paraphernalia, followed by transmission to blood donors through contaminated equipment, and later through heterosexual sex to partners of these groups and to and from female sex workers. More recently, HIV cases attributed to male‐male sex have come to predominate 10, 11, 12. By 2008, a nationwide survey found an overall HIV prevalence of 4.9% among men who have sex with men (MSM), placing this population as a top priority in China’s national HIV control strategy 13, 14.

Little is known about whether or not transgender women are impacted by HIV in China. Due to challenges with surveillance data, a scarcity of studies done specifically for the population, and difficulties in conducting probabilistic sampling, there is no national estimate of HIV among transgender women. As the case in many settings, data on transgender women are not distinguished in public health surveillance systems, or are merged with MSM, obscuring the burden of HIV and drivers of transmission specific to this population 1, 15, 16. The first reported prevalence of HIV infection in a population of Chinese transgender women, in 2014 by Cai *et al*., was 25.9%, significantly higher than their local MSM counterparts 17. The sample comprised only sex workers in the city of Shenyang. A subsequent survey of transgender women in 2016 in Shanghai and Tianjin, not restricted to sex workers, found HIV prevalence at 12.4% and 3.4% respectively 18. In this study, the sample was accrued through community‐based organizations, outreach at gay entertainment venues and sex work sites, and through gay social media.

We therefore set out to conduct a cross‐sectional survey of transgender women in two cities of Jiangsu province, China. The study used an adapted respondent‐drive sampling (RDS) approach 14, a recruitment method held to approximate probability sampling. The main objective of the survey was to measure HIV prevalence in a diverse community sample of Chinese transgender women. Secondarily, we explored risk factors for HIV acquisition as hypothesized in other studies and in our own formative research to help guide interventions for Chinese transgender women.

## 2 Methods

### 2.1 Study population, sampling design, and recruitment

Our cross‐sectional survey among transgender women was conducted from July 2018 to April 2019 in Nanjing and Suzhou cities of Jiangsu province, China. Selection of these two cities within our provincial jurisdiction was made on the basis of including the capital (Nanjing) and the major cultural city of Suzhou on the transport corridor to Shanghai. These cities were also endorsed by transgender community key informants as places where transwomen can be reached, who have social connections across each city and to the remainder of the province, could serve as initial seeds, and know each other well enough to refer other participants. Eligible participants were 18 years of age or older, lived in Jiangsu province, and had been assigned male sex at birth but currently self‐identified as a gender different from male. RDS methods were adapted to include a large number of seeds at the outset, flexibly add more seeds at different time points, more recruits per participant, and accrue recruitment across two different cities that were connected through the social networks of transgender women 14. Initial participants (seeds) were screened and referred by the Nanjing Transgender Women Shelter Center, the Suzhou Lesgo Group, and from key informants from the LGBT community who we contacted during a formative phase. After completing the survey, each seed was given 10 coupons to encourage recruiting up to 10 transgender women. These seeds were directed to select other eligible transgender women from their social networks through direct contact. Initially, 18 seeds (ten from Nanjing and eight from Suzhou) were enlisted who were diverse in terms of age, education, and occupation. The high number of seeds at the outset was chosen because key informants indicated that social networks were scattered and sizes tended to be small. After three months, the chains stemming from three of ten seeds in Nanjing and from six of eight seeds in Suzhou stopped recruitment. Therefore, new seeds were added to generate new chains of referrals. The new seeds were also chosen to be diverse with respect to age, education, and occupation. The second set of seeds included thirteen from Nanjing and seven from Suzhou. During recruitment, key variables were tracked to assess the stability of the composition of the sample in terms residence, education level and working at entertainment venues. An *a priori* sample size was set at 250 to provide reasonable precision on HIV prevalence (±5%) and sufficient power (80%) to identify significant associations with HIV infection (alpha = 0.05, effect sizes of OR > 1.7) in logistic regression analysis. Of the 273 initially screened, 21 were excluded as ineligible (including 19 identifying as male, 1 as ambigender and 1 as questioning) and two were excluded from analysis due to declining the HIV test.

The study site in Nanjing was at the HIV testing and counselling centre in the Jiangsu provincial Center for Disease Control and Prevention (CDC) and in Suzhou at the Rainbow community‐based organization office. Research team members were trained on the procedures of the survey, research ethics, HIV counselling and testing, and were sensitized to issues of gender identity, discrimination and stigma for the transgender population. Finger‐prick rapid tests were conducted to measure HIV prevalence. While awaiting results, participants completed a self‐administered electronic questionnaire on a mobile phone. Upon completion of the questionnaire, participants were counselled on their results and referrals to HIV care and other health and social services were made. Participants received a 200 RMB (approximately $30 USD) pre‐paid cellphone card. Those who recruited other eligible transgender women received an additional 100 RMB for each recruit.

The study was approved by the internal review boards of the University of California San Francisco and the Jiangsu provincial CDC. All participants provided written informed consent.

### 2.2 Measures

The study focuses on HIV prevalence and correlates of infection among transgender women. Socio‐demographic characteristics were measured as age, residence, marital status, education, employment and income. We asked if they had ever taken hormones to enhance gender presentation and if they ever had gender‐affirming surgeries, including removal of thyroid cartilage, breast augmentation, vaginoplasty, penectomy and orchiectomy. HIV‐related risk behaviours were measured by asking participants if they had ever engaged in sex work, the number of sexual partners in the last six months, alcohol or drug use before or during sex in the past 12 months, and any condomless insertive/receptive anal or vagina sex with the most recent partner in the last six months. Participants were also asked if they had tested for HIV in the last six months and if they had been diagnosed with an STI in the last 12 months.

HIV testing was done using a rapid assay (NewScen Coast Bio‐Pharmaceutical Co., Ltd., Tianjin, China). Participants with a positive result and not already in HIV care were referred to the local CDC for repeat and confirmatory testing, and results were provided back to our study. For those who self‐reported as HIV positive and in care and were confirmed by our rapid test, no further tests were conducted.

### 2.3 Statistical methods

Data were analysed using SPSS 20.0 version. Socio‐demographic characteristic, hormone use, gender‐affirming surgeries, HIV‐related risk behaviours and history of STI are described by frequencies and percentages in the survey sample. We examined HIV prevalence within groups defined by socio‐demographic characteristics and behaviours to identify potential risk and protective factors for HIV infection in bivariate analysis. Variables with differences in HIV prevalence in bivariate comparisons at *p *< 0.1, those thought *a priori* to be confounders, and variables whose inclusion in the model altered point estimates of significant predictors (even if not significant as primary effects) were consider as potential confounders and retained in multivariate logistic regression analysis. We repeated the analysis using RDS‐analyst to adjust for the sampling design, using the Gile’s estimator, also exporting individual weights to SPSS to conduct the bivariate and multivariate logistic regression analysis; these results are presented in the supplementary materials.

## 3 Results

Table 1 presents socio‐demographic characteristics, gender‐affirming surgeries and risk behaviours among participants. Of the 250 transgender women surveyed, 44.0% were between the ages of 18 and 24 years, 58.8% were permanent Jiangsu residents, 86.0% were never married and 28.8% had a university educational level or above. The majority (70.4%) reported being employed full time. A minority (20.8%) reported ever having engaged in sex work; somewhat more (39.2%) worked at entertainment venues. Nearly half (47.6%) had ever taken hormones and 18.0% had any gender‐affirming surgery, including removal of thyroid cartilage, breast augmentation, vaginoplasty, penectomy and orchiectomy. Over one third (38.4%) had two or more sexual partners in the last six months. Few (4.0%) reported using drugs before or during sex, such as rush, marijuana and synthetic cannabinoids, methamphetamine, prescription painkillers and crack. Self‐reported diagnosis of an STI in the past 12 months was reported by 6.4%.

**Table 1:** Socio‐demographic characteristics, sex work, gender‐affirming surgery and risk behaviours among transgender women, Jiangsu province, China, 2018 to 2019 (N=250)

Characteristic	N (%)
Age group (years, median 26, range 18 to 55)	
18 to 24	110 (44.0)
25 to 34	95 (38.0)
≥35	45 (18.0)
Official residence	
Jiangsu province	147 (58.8)
Other province	103 (41.2)
Marital status (i.e. legally to a woman)	
Single, never married	215 (86.0)
Married/cohabitating	13 (5.2)
Divorce/separated/widowed	22 (8.8)
Education	
High school or less	92 (36.8)
Technical college or some university	86 (34.4)
University degree or higher	72 (28.8)
Employment^a^	
Full‐time	176 (70.4)
Part‐time	20 (8.0)
Student/other	54 (21.6)
Monthly income (RMB, US $1 = approximately 6.8 RMB)	
≤2999	87 (34.8)
3000 to 4999	99 (39.6)
≥5000	64 (25.6)
Ever engaged in sex work	
Yes	52 (20.8)
No	198 (79.2)
Works at entertainment venue	
Yes	98 (39.2)
No	152 (60.8)
Ever taken hormones	
Yes	119 (47.6)
No	131 (52.4)
Ever had gender‐affirming surgery	
Yes	45 (18.0)
No	205 (82.0)
Number of sexual partners, last six months	
≤1	154 (61.6)
≥2	96 (38.4)
Insertive condomless anal sex, last six months	
Yes	19 (7.6)
No	231 (92.4)
Receptive condomless anal sex, last six months	
Yes	55 (22.0)
No	195 (78.0)
Insertive condomless vaginal sex, last six months	
Yes	9 (3.6)
No	241 (96.4)
Receptive condomless vaginal sex, last six months (the “yes” respondents reported vaginoplasty)	
Yes	6 (2.4)
No	244 (97.6)
Drug use before or during sex, last 12 months	
Yes	10 (4.0)
No	240 (96.0)
Had an STI diagnosis in the last 12 months	
Yes	16 (6.4)
No	234 (93.6)

a Full time job means employed by one company or organization with fixed, full hours. Part time job means employed by multiple organizations with flexible working hours and places. Students are treated as a separate category regardless of full or part time work.

The prevalence of HIV among transgender women in our survey was 14.8 (95% CI 10.6, 19.8) (Table 2). Of the 37 testing HIV‐positive, 28 (75.6%) reported being previously diagnosed. In multivariable analysis, significant correlates of HIV infection were age 25 to 34 years (adjusted odds ratio (AOR) 3.52, 95% CI 1.23, 10.13) and ≥35 years (AOR 3.56, 95% CI 1.05, 12.06), work at entertainment venues (AOR 2.48, 95% CI 1.01, 6.09), never taking hormones (AOR 3.22, 95% CI 1.24, 8.35) and diagnosed with an STI in the last 12 months (AOR 9.76, 95% CI 2.86, 33.27).

**Table 2:** Bivariate and multivariate correlates of HIV prevalence, socio‐demographic characteristics, gender‐affirming surgery, and risk behaviours among transgender women, Jiangsu province, China, 2018 to 2019 (N=250)

Characteristic	HIV prevalence, n (%)	OR (95% CI), *p*	AOR^a^ (95% CI), *p*
Total	37 (14.8)	–	–
Age group (years)			
18 to 24	6 (5.5)	–	–
25 to 34	20 (21.1)	4.62 (1.77, 12.07), 0.002	3.52 (1.23, 10.13), 0.019
≥35	11 (24.4)	5.61 (1.93, 16.31), 0.002	3.56 (1.05, 12.06), 0.041
Official residence			
Jiangsu province	25 (17.0)	–	–
Other province	12 (11.7)	1.55 (0.74, 3.26), 0.243	–
Marital status			
Single, never married	30 (14.0)	–	–
Married/cohabitating	3 (23.1)	1.85 (0.48, 7.11), 0.371	–
Divorced/separated/widowed	4 (18.2)	1.37 (0.43, 4.33), 0.591	–
Education			
High school or less	23 (25.0)	–	–
Some technical college, university	10 (11.6)	0.40 (0.18, 0.89), 0.025	0.84 (0.31, 2.27), 0.733
University degree or higher	4 (5.6)	0.18 (0.06, 0.54), 0.002	0.57 (0.15, 2.20), 0.416
Monthly income (RMB, approximately 6.8 RMB/$1)			
≤2999	11 (12.6)	–	–
3000 to 4999	14 (14.1)	1.14 (0.49, 2.66), 0.765	–
≥5000	12 (18.8)	1.59 (0.65, 3.89), 0.305	–
Had ever engaged in sex work			
Yes	10 (19.2)	–	–
No	27 (13.6)	0.66 (0.30, 1.48), 0.314	–
Works at entertainment venue			
No	12 (7.9)	–	–
Yes	25 (25.5)	4.00 (1.90, 8.41), <0.001	2.48 (1.01, 6.09), 0.047
Had ever taken hormones			
Yes	7 (5.9)	–	–
No	30 (22.9)	4.75 (2.00, 11.29), <0.001	3.22 (1.24, 8.35), 0.016
Ever had gender‐affirming surgery			
Yes	6 (13.3)	–	–
No	31 (15.1)	1.16 (0.45, 2.97), 0.760	–
Number of sexual partners, last six months			
≤1	23 (14.9)	–	–
≥2	14 (14.6)	0.97 (0.47, 2.00), 0.939	–
Insertive condomless anal sex, last six months			
Yes	2 (10.5)	–	–
No	35 (15.2)	1.52 (0.34, 6.86), 0.588	–
Receptive condomless anal sex, last six months			
Yes	11 (20.0)	–	–
No	26 (13.3)	0.62 (0.28, 1.34), 0.222	–
Insertive condomless vagina sex, last six months			
Yes	1 (11.1)	–	–
No	36 (14.9)	1.41 (0.17, 11.57), 0.752	–
Receptive condomless vagina sex, last six months			
Yes	1 (16.7)	–	–
No	36 (14.8)	0.87 (0.10, 7.63), 0.896	–
Drug use before or during sex, last 12 months			
Yes	3 (23.1)	–	–
No	34 (14.3)	0.56 (0.15, 2.13), 0.394	–
Had an STI diagnosis, last 12 months			
No	26 (11.1)	–	–
Yes	11 (68.8)	17.60 (5.67, 54.65), <0.001	9.76 (2.86, 33.27), <0.001

a Adjusted for other variables in the model.

## 4 Discussion

The prevalence of HIV found in our study places transgender women in Nanjing and Suzhou close to the international estimate 1. Our figure of 14.8% (95% CI 10.4 to 19.8) is statistically consistent with the pooled estimate of 17.7% for HIV prevalence among transgender women in low‐ to middle‐ income countries 1. The prevalence is notably higher than that of most other key populations at risk for HIV in China 10, 11, 12, 13, 14, with rare exceptions in the southwest region of the country 13. Moreover only three‐fourths of transgender women testing positive in our study knew their HIV status – a level that falls well below the UNAIDS target of 90% awareness 19.

Our study also identified correlates of HIV infection that can help target the response and identify potential risk factors for infection among transgender women in China. Older age was associated with higher prevalence of HIV. Although greater than 5% of transgender youth age 18 to 24 years were already HIV positive, acquisition of infection appeared to rapidly accelerate to 3.5‐fold by the late 20s and early 30s. This finding is similar to those of studies in Cambodia and Thailand 4, 20 and consistent with a cumulative probability of being exposed to HIV over the life course 21. The particularly steep rise in HIV prevalence from 25 to 35 may point to a critical period to intervene vigorously with behavioural and biological interventions, such as those being delivered by the “China AIDS Fund for Non‐Governmental Organization” (CAFNGO) 22. Although no correlation was found between marital status and HIV infection in the study, far fewer were or had been married than would be expected for this age group. In the Chinese context, transgender women may have conflicts with family due to parents’ desire for their child to get married and have children to continue the blood line. This conflict may result in rejection and isolation by family and society, which may exacerbate transgender women’s vulnerability and risk for HIV infection 5.

While the body of literature finds sex work associated with elevated HIV risk 17, 23, 24, we did not find such a direct association in our study. We suspect a likely response bias of under‐reporting because sex work is illegal in China 11, as in much of the world. Meanwhile, reporting work in an entertainment centre imparted a 2.5‐fold increased risk of HIV infection. Entertainment centres may be indirect sex work venues for transgender women, as is common in Thailand 25. Because sex work is criminalized in China, participants may have been more comfortable responding that they worked as employees of these entertainment centres rather than reporting engagement in sex work. Transgender women who work in entertainment centres may also be at greater risk of HIV because they have increased access to male sexual partners than transgender women who are more hidden 24. Entertainment centres may therefore be effective locations for outreach and as sites for tailored interventions for transgender women at elevated risk for HIV.

Lack of use of hormones was also associated with a several‐fold increased odds of HIV infection. Under half of transgender women in our survey had ever taken hormones and fewer than one in five had ever had gender‐affirming surgery, figures that are quite low compared with those of other countries 3, 9, 26. At least one study in China documented a large unmet need and desire for gender transition care, including 90% demand for hormones and 50% demand for gender‐affirming surgery 27. The reason for the association of non‐hormone use and HIV is not clear from our data. In other settings, lack of hormone use was associated with specific gender identities, such as “travesti” in Rio de Janeiro 28 and “transsexual” in San Francisco 29, which in turn were groups with higher prevalence of HIV. These groups of transgender women may not be on feminizing hormone therapy because they cannot afford or do not desire the feminizing effects of oestrogen, thus transgressing gender norms in the trans and cisgender women communities. This transgression may result in higher overall vulnerability and thus greater risk for HIV. Transgender women who did sex work may have chosen not to use hormones for fear of a reduced libido and erectile function. As described above, transgender women who do sex work may have had more sexual partners and therefore higher sexual risk for HIV than transgender women who did not do sex work. Non‐hormone use may also be a marker of being extremely hidden and disconnected from the trans community and/or fearful of engaging in medical transition. Thus, discrimination from economic opportunities and isolation within and outside of community may be the drivers of HIV risk for transgender women not on hormones. Barriers to economic opportunities, integration in society and access to professional medical transition care therefore need to be addressed to reduce HIV risk and to improve psychological wellbeing and quality of life for Chinese transgender women.

Not surprising, history of STI diagnosis was associated with HIV infection, consistent with transgender health research worldwide 4, 9 and with the common sexual mode of transmission. The prevalence of STI may be under‐reported considering the context of the question was having a recent diagnosis, many infections being asymptomatic, and potential under‐recognition of pharyngeal and rectal sites of infection by respondents and clinicians. Increased extra‐urethral screening and sensitization of providers and the community should be implemented in HIV and STI programmes tailored for transgender women.

Our study has limitations. While our RDS recruitment approach successfully met the sample size and the composition of the sample appeared diverse with respect to demographic characteristics, there is no census for transgender women against which to gauge representation. Generalization of findings to all transgender women in the two cities of Jiangsu province, let alone elsewhere in China, must be cautious. As in every country of the world, there is transgender‐related stigma and discrimination in healthcare settings that can affect participation. We also recognize the sensitivity of questions, including those on illegal behaviours, may result in bias with under‐estimation of factors such as sex work and drug use. Moreover we acknowledge that our brief survey did not allow for extensive detail in many areas, which will need to be evaluated in more depth in larger, longitudinal and qualitative studies. For example, partner‐by‐partner information would help elucidate potential sources of HIV infection to transgender women.

## 5 Conclusions

Our study demonstrated a high prevalence of HIV among transgender women in two cities in Jiangsu province, China. The prevalence of 14.8% is the highest measured in any population in this part of eastern China. Higher prevalence was observed among transgender women who were older, were working at entertainment venues, had no history of hormone use, and a history of STI. Access to gender‐affirming care was low. These data support policies to prioritize transgender women for HIV testing efforts, for research to better understand the specific drivers of infection in this population, and for creating trans‐friendly HIV/STI care and prevention programmes.

## Competing interests

None.

## Authors’ contributions

HY, EW and WM designed the study and provided scientific oversight. WX, YC and YC conducted informed consent, interviews and data management. JL and ZY contributed to the design of the measures and interpretation of findings. HY conducted the primary analysis. HY, WM and EW drafted the manuscript and incorporated authors’ comments. All authors provided critical review and edits. The final manuscript for submission was approved by all authors.

## Funding

This study was supported by the National Institute on Minority and Health Disparities (R01MD010678).

## Supporting information


**Table S1. **RDS design‐adjusted socio‐demographic characteristics, sex work, gender‐affirming surgery and risk behaviours among transgender women, Jiangsu province, China, 2018 to 2019 (N = 250)
**Table S2. **RDS design‐adjusted bivariate and multivariate correlates of HIV prevalence, socio‐demographic characteristics, gender‐affirming surgery and risk behaviours among transgender women, Jiangsu province, China, 2018 to 2019 (N = 250)
