# Supplementary material for: High HIV prevalence and associated risk factors among transgender women in China: a cross‐sectional survey
Source: J Int AIDS Soc. 2019 Nov 15;22(11):e25417. doi: 10.1002/jia2.25417 (PMC6856602; doi:10.1002/jia2.25417)
Supplement: Supplementary file 1 — Table S1. RDS design‐adjusted socio‐demographic characteristics, sex work, gender‐affirming surgery and risk behaviours among transgender women, Jiangsu province, China, 2018 to 2019 (N = 250) Table S2. RDS design‐adjusted bivariate and multivariate correlates of HIV prevalence, socio‐demographic characteristics, gender‐affirming surgery and risk behaviours among transgender women, Jiangsu province, China, 2018 to 2019 (N = 250) [file JIA2-22-e25417-s001.docx]

**Supplementary materials:** RDS design-adjusted estimates

**Table 1. RDS design-adjusted socio-demographic characteristics, sex work, gender-affirming surgery, and risk behaviors among transgender women, Jiangsu province, China, 2018-19 (N=250)**

| **Characteristic** | **Crude %**  **(n)** | **RDS-adjusted population estimates %**  **(95% CI)^a^** |
| --- | --- | --- |
| Age group (years) (median 26, range 18-55 years) |  |  |
| 18-24 | 44.0 (110) | 60.3 (49.7 –69.0) |
| 25-34 | 38.0 (95) | 27.2 (20.8 – 35.0) |
| ≥35 | 18.0 (45) | 12.5 (7.7 – 18.6) |
| Official residence |  |  |
| Jiangsu province | 58.8 (147) | 61.9 (54.1 – 70.4) |
| Other province | 41.2 (103) | 38.1 (29.6 – 45.9) |
| Marital status (i.e., legally to a woman) |  |  |
| Single, never married | 86.0 (215) | 90.3 (86.3 – 94.3) |
| Married/cohabitating | 5.2 (13) | 3.4 (1.8 – 5.6) |
| Divorce/separated/widowed | 8.8 (22) | 6.2 (2.7 – 9.7) |
| Education |  |  |
| High school or less | 36.8 (92) | 34.1 (26.3 – 43.9) |
| Technical college or some university | 34.4 (86) | 34.2 (25.7 – 40.2) |
| University degree or higher | 28.8 (72) | 31.8 (22.3 – 42.3) |
| Employment^b^ |  |  |
| Full-time | 70.4 (176) | 63.0 (59.7 – 75.7) |
| Part-time | 8.0 (20) | 3.9 (1.5 – 5.5) |
| Student/other | 21.6 (54) | 33.1 (21.0 – 36.9) |
| Monthly income (RMB, US $1 = ~6.8 RMB) |  |  |
| ≤2,999 | 34.8 (87) | 41.9 (31.0 – 46.7) |
| 3,000-4,999 | 39.6 (99) | 40.5 (34.6 – 49.7) |
| ≥5,000 | 25.6 (64) | 17.5 (13.4 – 25.4) |
| Had ever engaged in sex work  Yes  No | 20.8 (52)  79.2 (198) | 18.4 (12.6 – 23.9)  81.6 (76.1 – 87.4) |
| Works at entertainment venue |  |  |
| Yes | 39.2 (98) | 30.4 (23.0 – 40.4) |
| No | 60.8 (152) | 69.6 (59.6 – 77.1) |
| Had ever taken hormones |  |  |
| Yes | 47.6 (119) | 44.6 (32.8 – 52.9) |
| No | 52.4 (131) | 55.4 (47.1 – 67.2) |
| Ever had gender-affirming surgery |  |  |
| Yes | 18.0 (45) | 13.8 (9.1 – 19.2) |
| No | 82.0 (205) | 86.2 (80.9 – 90.9) |
| Number of sexual partners, last 6 months  ≤1  ≥2 | 61.6 (154)  38.4 (96) | 64.3 (55.1 – 71.9)  35.7 (28.1 – 44.9) |
| Insertive condomless anal sex, last 6 months  Yes  No | 7.6 (19)  92.4 (231) | 7.8 (4.0 – 12.1)  92.2 (87.9 – 96.1) |
| Receptive condomless anal sex, last 6 months  Yes  No | 22.0 (55)  78.0 (195) | 17.3 (11.7 – 21.3)  82.7 (78.7 – 88.3) |
| Insertive condomless vagina sex, last 6 months  Yes  No | 3.6 (9)  96.4 (241) | 5.1 (2.1 – 8.4)  94.9 (91.6 – 97.9) |
| Receptive condomless vagina sex, last 6 months (the “yes” respondents reported vaginoplasty)  Yes  No | 2.4 (6)  97.6 (244) | 1.6 (0.4 – 2.8)  98.4 (97.2 – 99.6) |
| Drug use before or during sex, last 12 months  Yes  No | 4.0 (10)  96.0 (240) | 4.6 (1.1 – 7.5)  95.4 (92.5 – 99.0) |
| Had an STI diagnosis in the last 12 months  Yes  No | 6.4 (16)  93.6 (234) | 3.4 (1.5 – 6.8)  96.6 (93.2 – 98.6) |

^a^Confidence intervals computed using the Gile’s bootstrap method from RDS-Analyst.

^b^Full time job means employed by one company or organization with fixed, full hours. Part time job means employed by multiple organizations with flexible working hours and places. Students are treated as a separate category regardless of full or part time work.

**Table 2. RDS design-adjusted bivariate and multivariate correlates of HIV prevalence, socio-demographic characteristics, gender-affirming surgery, and risk behaviors among transgender women, Jiangsu province, China, 2018-19 (N=250)**

| **Characteristic** | **HIV prevalence**  **n (%)** | **RDS-weighted**  **OR (95% CI), p** | **RDS-weighted**  **AOR* (95% CI), p** |
| --- | --- | --- | --- |
| Total | 37 (14.8) |  |  |
| Age group (years) |  |  |  |
| 18-24 | 6 (5.5) | - | - |
| 25-34 | 20 (21.1) | 2.20 (0.81, 5.95), 0.121 | 2.11 (0.65, 6.80), 0.213 |
| ≥35 | 11 (24.4) | 3.40 (1.10, 10.46), 0.033 | 2.61 (0.66, 10.32), 0.172 |
| Official residence  Jiangsu province  Other province | 25 (17.0)  12 (11.7) | 1.84 (0.73, 4.64), 0.195 |  |
| Marital status |  |  |  |
| Single, never married | 30 (14.0) | - |  |
| Married/cohabitating | 3 (23.1) | 1.45 (0.19, 10.87), 0.72 |  |
| Divorce/separated/widowed | 4 (18.2) | 0.84 (0.14, 5.07), 0.85 |  |
| Education |  |  |  |
| High school or less | 23 (25.0) | - | - |
| Technical college or some  university | 10 (11.6) | 0.35 (0.12, 1.03), 0.056 | 0.88 (0.23, 3.36), 0.854 |
| University degree or higher | 4 (5.6) | 0.43 (0.15, 1.23), 0.116 | 1.27 (0.32, 4.98), 0.733 |
| Monthly income (RMB, US $1 = ~6.8 RMB) |  |  |  |
| ≤2,999 | 11 (12.6) | - |  |
| 3,000-4,999 | 14 (14.1) | 0.63 (0.23, 1.70), 0.361 |  |
| ≥5,000 | 12 (18.8) | 1.04 (0.36, 3.01), 0.945 |  |
| Had ever engaged in sex work |  |  |  |
| Yes | 10 (19.2) | - |  |
| No | 27 (13.6) | 0.69 (0.25, 1.89), 0.467 |  |
| Works at entertainment venue |  |  |  |
| No | 12 (7.9) | - | - |
| Yes | 25 (25.5) | 3.42 (1.43, 8.20), 0.006 | 1.85 (0.61, 5.56), 0.275 |
| Had ever taken hormones |  |  |  |
| Yes | 7 (5.9) | - | - |
| No | 30 (22.9) | 8.56 (2.32, 31.61), 0.001 | 5.92 (1.42, 24.61), 0.015 |
| Ever had gender-affirming surgery |  |  |  |
| Yes | 6 (13.3) | - |  |
| No | 31 (15.1) | 2.92 (0.53, 16.10), 0.218 |  |
| Number of sexual partners, last 6 months |  |  |  |
| ≤1 | 23 (14.9) | - |  |
| ≥2 | 14 (14.6) | 0.72 (0.28, 1.82), 0.487 |  |
| Insertive condomless anal sex, last 6 months |  |  |  |
| Yes | 2 (10.5) | - |  |
| No | 35 (15.2) | 1.71 (0.27, 10.94), 0.572 |  |
| Receptive condomless anal sex, last 6 months |  |  |  |
| Yes | 11 (20.0) | - | - |
| No | 26 (13.3) | 0.38 (0.15, 0.96), 0.040 | 0.63 (0.20, 2.05), 0.444 |
| Insertive condomless vagina sex, last 6 months |  |  |  |
| Yes | 1 (11.1) | - |  |
| No | 36 (14.9) | 2.21 (0.12, 40.16), 0.591 |  |
| Receptive condomless vagina sex, last 6 months |  |  |  |
| Yes | 1 (16.7) | - |  |
| No | 36 (14.8) | 2.06 (0.04, 121.98), 0.729 |  |
| Drug use before or during sex, last 12 months |  |  |  |
| Yes | 3 (23.1) | - |  |
| No | 34 (14.3) | 1.13 (0.15, 8.33), 0.906 |  |
| Had an STI diagnosis in the last 12 months |  |  |  |
| No | 26 (11.1) | - | - |
| Yes | 11 (68.8) | 56.08 (10.33, 304.45), <0.001 | 30.72 (4.93, 191.57), <0.001 |

*Adjusted for other variables in the model.
